# Irritable Bowel Syndrome and Gastrointestinal Parasite Infection in a Developing Nation Environment

**DOI:** 10.1155/2012/343812

**Published:** 2012-02-19

**Authors:** Douglas R. Morgan, Matthew Benshoff, Mercedes Cáceres, Sylvia Becker-Dreps, Loreto Cortes, Christopher F. Martin, Max Schmulson, Rodolfo Peña

**Affiliations:** ^1^Division of Gastroenterology and Hepatology, School of Medicine, University of North Carolina at Chapel Hill, Bioinformatics Building, Suite 4143, 130 Mason Farm Road, Chapel Hill, NC 27599-7080, USA; ^2^School of Medicine, Universidad Nacional Autónoma de Nicaragua (UNAN), León, Nicaragua; ^3^School of Medicine, Universidad Nacional Autónoma de México (UNAM), Hospital General de México, Mexico City, DF, Mexico; ^4^Centro de Intervención e Intervenciones en Salud, León, Nicaragua

## Abstract

Postinfectious IBS is defined in the industrialized world as IBS onset following a sentinel gastrointestinal infection. In developing nations, where repeated bacterial and parasitic gastrointestinal infections are common, the IBS pathophysiology may be altered. Our aim was to investigate the relationship between intestinal parasite infection and IBS in the “nonsterile” developing world environment. IBS subjects were identified from a population-based sample of 1624 participants using the Rome II Modular Questionnaire. Stool samples from cases and randomly selected controls were examined for ova and parasites. Logistic regression models explored the relationship between IBS and parasite infection. The overall IBS prevalence among participants was 13.2% (9.3% males, 15.9% females). There was no difference in parasite carriage between IBS cases and controls, 16.6% versus 15.4% (*P* = 0.78), nor among IBS subtypes. The pathophysiology of post-infectious IBS may be altered in the developing world as compared to industrialized nations and warrants investigation.

## 1. Introduction

Irritable bowel syndrome (IBS) is a functional gastrointestinal disorder which affects approximately 12% of persons globally [1, 2]. Several studies have examined the prevalence of IBS in different geographic regions, and in general, have found the prevalence of IBS to be higher in industrialized nations and lower in developing nations [3, 4]. This may be due to differences in diagnostic criteria and their translations, health care access and use, and other factors which differ between countries [4]. In his review, Kang suggests additional differences in host genetics, diet, and health belief models may contribute to the variability in the prevalence of IBS between countries [4]. Also in this review, the paucity of IBS investigations from Latin America is highlighted.

 Postinfectious IBS (PI-IBS) is defined as the onset of IBS following a sentinel gastrointestinal infection, particularly infectious gastroenteritis [5, 6]. The prevalence of PI-IBS following infectious gastroenteritis ranges from 4% to 31%, with a pooled incidence of 10% [7, 8]. PI-IBS has been predominantly described for residents of the industrialized world in two scenarios: traveler's diarrhea and gastroenteritis outbreaks. Following these intestinal infections, there is a higher prevalence of diarrhea-predominant IBS (IBS-D) symptoms. Multiple pathogens have been implicated in the development of IBS in the so-called “sterile” environments of developed nations, including *E. coli, C. jejuni, *and *S. sonnei *[9–12]. Not all studies support a causative role of infectious gastroenteritis [13]. A number of nonbacterial pathogens have also been implicated in PI-IBS, including viral and parasitic organisms [14–17]. Several parasites including *E. histolytica*, *Giardia *spp., *B. hominis, *and *Trichinella *spp. have been discussed as contributing factors to the development of IBS, though the relationship is less well defined [14–21]. 

 While the precise pathophysiology of PI-IBS is unknown, suggested factors include altered serotonin signaling activity, inflammation, malabsorption, and small intestinal bacterial overgrowth [10–12, 22]. Although it is apparent that the majority of gastrointestinal infections do not result in IBS, the incidence of IBS after infection may provide insight into the etiology of the disease and the “hygiene hypothesis” [23]. 

The effect of gastrointestinal infections on the development of IBS is largely unexplored in the developing world. The developing world environment may be considered “nonsterile,” with the majority of the population exposed to intermittent acute infections beginning in infancy, and a subset with chronic gastrointestinal infections [24, 25]. For example, in Mexico, the overall prevalence of infection with *E. histolytica/E. dispar* in pregnant women has been estimated to be between 22 and 35%, with 15% of their children also infected [26]. The concept of the sentinel infection to trigger PI-IBS may not be applicable and, in addition, would be difficult to ascertain.

 We examined the relationship between IBS and parasite infection using a population-based case-control design in the “nonsterile” developing nation environment in western Nicaragua. In this setting, parasite carriage may serve as a surrogate for repeated exposures to gastrointestinal pathogens and infection. As described below, the unique Health and Demographic Surveillance Site in León, Nicaragua (HDSS-León) was used to select a random population-based sample for identification of IBS cases and healthy controls.

## 2. Materials and Methods

### 2.1. Setting

The Center for Epidemiology and Health (CIDS) within the University of Nicaragua, León (UNAN-León) maintains HDSS-León for the region with a sampled population of over 200,000. HDSS-León, established by CIDS in 1993, includes approximately 11,000 households and 55,000 individuals, or 30% of the population of León municipality [27]. This is the only Latin American member of the INDEPTH network, an international network of demographic surveillance population cohorts in developing nations [28]. The region's population is Hispanic Mestizo ethnic origin, with less than 10% from indigenous groups. Half of the population is under the age of 15. Nicaragua consistently ranks among the poorest countries in Latin America, with a per capita gross national income of US$1000 [29]. In addition, parasite infection is known to be high in the León province of western Nicaragua [25, 30]. 

### 2.2. Study Design

The study utilized a population-based, nested case-control design with household interviews. The cross-sectional survey of the functional gastrointestinal disorders was performed with randomly selected individuals (*n* = 1624) within the HDSS-León. IBS cases were identified using the Rome II Modular Questionnaire that has been previously translated into Spanish and validated for Mexico and Central America [31, 32]. IBS cases were further characterized using the Rome II criteria as IBS-D, constipation-predominant (IBS-C), and alternating/mixed (IBS-A), for those not fulfilling criteria for either of the above categories. Within this cross-sectional survey (*n* = 1.642), we conducted a nested case-control study of IBS cases and healthy controls (*n* = 359). Healthy controls without IBS were randomly selected from the cross-sectional survey participants in a 1 : 1 ratio for stool collection and related study data. Cases and controls were interviewed by study team physicians.

Socioeconomic status was assessed using a validated poverty index which was calculated using the United Nation's unsatisfied basic needs measurement, based on housing, sanitation, education, and employment [33–35] and validated in Nicaragua [36, 37]. Specific factors related to the environment that were examined included water source, toilet or latrine use, household construction, and neighborhood.

### 2.3. Laboratory Analysis

Laboratory stool analysis was performed in the UNAN-León Center for Infectious Disease Research, by methods previously described [38]. The stool specimens were collected in labeled plastic vials without preservatives and examined in less than two hours. Stool samples were subjected to macroscopic examination, to check the consistency and to evaluate for the presence of blood, mucus, or adult helminth parasites. Specimens were examined by direct microscopy with saline and iodine by microbiologists. In addition to direct microscopy of fresh smears, formalin ethyl acetate sedimentation technique was used for detection of cysts and eggs, iron hematoxylin staining for amebas and flagellates, and modified Ziehl-Neelsen staining for detection of enteric coccidia. No additional tests for viral and bacterial infections were performed. These university-based microscopy methods have been shown to be highly reproducible in Nicaragua, and with comparable sensitivity to PCR methodology for detection of *Entamoeba *spp. [38]. 

Parasites were classified as either pathogenic or commensal, based upon internationally accepted classification [39]. The *E. histolytica/E. dispar *complex was classified as commensal for the primary analysis, because the prevalence of *E. dispar *exceeds that of *E. histolytica *(5 : 1) in Nicaragua [38]. We further performed a sensitivity analysis classifying either of these amoebas instead as pathogenic. Since no difference was found in the prevalence of the *E. histolytica/E. dispar *complex between IBS and controls (see Section 3), the molecular differentiation of the two species was not performed. Subjects with pathogenic parasitic infection on stool exam were offered treatment with antiparasitic medications per the local standard of care.

### 2.4. Statistical Analysis

Data was analyzed using SPSS version 12.0 statistical software (Chicago, IL, USA). Mantel-Haenszel odds ratios were calculated to determine the association between IBS and parasite infection. Possible confounding factors, including age, gender, poverty index, household water source, latrine or toilet use, household construction, and neighborhood, were examined. The current study was approved by the institutional review boards of the University of North Carolina, Chapel Hill and UNAN-León.

## 3. Results

### 3.1. Characteristics of the Participants

The overall prevalence of IBS in the population-based cross sectional study was 13.2%, with the prevalence of 15.9% in females and 9.3% in males. The IBS subsets were nearly evenly distributed: IBS-D 25%, IBS-C 32%, and IBS-A 43%. The characteristics of the study population (*n* = 357) are presented in Table 1. The median age was 39.0 years old with a range of 18 to 66. In the households, 68% had their basic needs met, 28% lived in poverty, and 4.0% lived in extreme poverty. Nearly all subjects lived in moderate-to-severe poverty by developed nation standards. Forty-one percent used latrines or did not have household sanitary facilities.

### 3.2. Association between IBS and Parasite Infection

The overall prevalence of parasitic infection in the nested case-control study population was 16.0%. Of the 214 identified IBS cases, 163 stool samples adequate for examination were obtained. Evaluable stool samples were obtained from 194 controls. No statistically significant association was found between the presence of parasites upon stool examination and the presence of IBS. Specifically, 16.6% of IBS cases and 15.4% of controls tested positively for parasite infection. Those with IBS had the same odds of parasitic infection as the controls (OR = 1.09, 95% CI 0.62, 1.91). Furthermore, when broken down by individual parasites, none showed a significant association with IBS, having a range of ORs from 0.75 (95% CI 0.36, 1.57) to 6.12 (95% CI 0.71, 52.8) (Table 2). Lastly, we found no association between parasite infection and IBS in the IBS subgroups: IBS-D (OR = 0.73, 95% CI 0.21, 1.99) and IBS-C (OR = 1.46, 95% CI 0.63, 3.16).

When the parasites were classified into the pathogenic or commensal groups, neither of the two groups showed an association with IBS prevalence (Table 2). As noted, differentiation of *E. histolytica* and *E. dispar* was not performed, and the *E. histolytica/E. dispar* complex was classified as commensal. A sensitivity analysis, classifying the *E. histolytica/E. dispar* complex as pathogenic, also resulted in no association between parasite infection and IBS in either the pathogenic or commensal groups.

 In addition to parasite presence, other possible confounding factors were analyzed including water source, sanitation (toilet or latrine use), household construction, neighborhood, and socioeconomic status (poverty index). There were no statistically significant differences between IBS cases and controls for water source, sanitation, housing construction, neighborhood, or overall poverty index; therefore, these factors were not included in the final logistic regression model.

## 4. Discussion

This study found a lack of an association between IBS and parasite infection in the developing nation environment of Nicaragua, Central America. This is the one of first studies to utilize a population-based sampling frame and Rome criteria assessment to examine the phenomenon of IBS in a “nonsterile” developing population, where repeated exposure to gastrointestinal pathogens is common. The overall prevalence of IBS based upon the Rome II criteria in our population-based study (*n* = 1.642) was 13.2%. This is comparable to the prevalence reported in the community-based study conducted in Mexico (16.0%) and in other countries as reported by Kang [4, 40]. Importantly, the current study identified IBS cases from the community, rather than from a health care setting, potentially removing selection bias and related confounding factors that may be associated with access and health care seeking behaviors. We found a similar prevalence of parasite infection between IBS cases and controls suggesting that gastrointestinal infection and specifically, parasite infection, is unlikely to be a significant attributable risk factor to the development of IBS.

 Our findings of a 20.2% prevalence of *Entamoeba *spp., 3.0% prevalence of *G. lamblia,* 0.6% prevalence of *A. lumbricoides,* and 1.8% prevalence of *T. trichiura* among IBS cases are in agreement with other regional studies of parasite prevalence, including prior investigations in Nicaragua [25]. A study from Guatemala reported a 2.7% prevalence of *G. lamblia* and a similar prevalence for the combination of *T. solium*, *A. lumbricoides, *and* T. trichuria* in IBS [41]. However, our study differed from a study in Pakistan, which found a higher prevalence of *B. hominis *and* D. fragilis *among IBS cases as compared to controls [42]. A similar study from Thailand noted prevalent *B. hominis* infection, yet no difference between IBS subjects and controls (13.6% versus 20%; *P* = 0.87) [43]. Interestingly, in an investigation in Pakistan, wherein a cohort of IBS subjects was followed over 48 weeks with monthly stool exams, spontaneous clearance of *E. histolytica* was noted in a subset (4/22) yet without symptom improvement [44].

 In industrialized nations, emerging evidence suggests that PI-IBS may account for a measureable percentage of the total burden of IBS in the community [7, 8]. While the specifics of the pathogen, severity, and duration of the infection play a role, a range of host factors are also responsible for the initiation and maintenance of IBS following enteritis. Host factors include host susceptibility genotypes, inflammatory response, and the psychosocial dynamic. In developing nations where water supplies, sanitation, and food hygiene are compromised, repeated episodes of gastroenteritis and chronic gastrointestinal infection begin in infancy. A segment of the IBS population may have the equivalent of PI-IBS by the western model, but without a “sentinel infection.” Alternatively, the pathophysiology model for IBS in developing nations may have a different balance of factors. In fact, per the hygiene hypothesis, gastrointestinal pathogen exposure in childhood may provide an element of immune tolerance and/or be protective and preclude the development of IBS per the western model [23]. Preliminary data from an ongoing multinational internet survey is also supportive: 21% of IBS patients in North America and Northern Europe may be considered “postinfectious” as compared to 14% in the rest of the world [45]. It is clear that chronic colonization by pathogens and commensals, which are prevalent infections in tropical environments, serves to regulate gastrointestinal inflammation and the immune response. Important examples include helminth colonization and ulcerative colitis [46], and *H. pylori* and asthma [47]. Similar studies investigating the relationship between parasites and IBS are needed in the northern hemisphere.

 The current study was strengthened by the use of a population-based study design with household interviews, which helps to address issues of selection and reporting bias present in clinic-based samples. In addition, Nicaragua is an appropriate setting for the study, as it consistently ranks among the poorest countries in Latin America. In spite of the large initial community sample, our investigation may not have been sufficiently powered to detect a difference in infection prevalence and further study may be warranted. Our study may have been limited by the focus upon parasitic rather than bacterial infections. Given the exposure to multiple episodes of all forms of gastroenteritis and health system access issues for the majority of subjects, recall bias would have precluded true assessment of past bacterial infections. In addition, it is suggested that parasite carriage is a reasonable surrogate for exposure to gastrointestinal pathogens in this setting. Lastly, parasites were classified as commensal or pathogen based on their ability to cause clinical disease. As noted, the characterization of intestinal parasites did not include the differentiation of *E. histolytica* and *E. dispar,* although this did not appear to be a factor based upon our sensitivity analysis.

## 5. Conclusions

In this population-based study, a significant difference was not observed in the prevalence of intestinal parasite infection among patients with IBS compared with healthy controls in the developing nation setting of Nicaragua. The reality of frequent gastrointestinal infections, and the lack of a “sentinel infection,” may suggest an alternate PI-IBS pathophysiology model in this setting and warrants investigation.

## Figures and Tables

**Table 1:** Summary of the study population.

	IBS cases (*N* = 163)	Controls (*N* = 194)	*P* value	Total (*N* = 357)
Gender				
Male Female	24.5% (40) 75.4% (123)	31.4% (61) 68.6% (133)	0.16	28.3% (101) 71.7% (256)

Age (in years)				
18–34 35–54 55–66	39.9% (65) 47.2% (77) 12.9% (21)	37.6% (73) 49.7% (98) 11.8% (23)	0.83	38.7% (138) 49.0% (175) 12.3% (44)

Household water source				
Municipal supply^†^ Other	97.5% (159) 2.5% (4)	97.4% (189) 2.6% (5)	0.98	97.5% (348) 2.5% (9)

Sanitary conditions				
Toilet Latrine or none	58.2% (95) 41.7% (68)	60.3% (117) 39.6% (77)	0.85	59.4% (212) 40.6% (145)

Poverty index				
Basic needs met basic needs unmet	63.8% (104) 36.2% (59)	71.1% (138) 28.9% (56)	0.20	67.8% (242) 32.2% (115)

^†^Municipal water source refers to water delivery to the household or nearby community source for at least 4 hours per day.

**Table 2:** Parasite carriage among IBS cases and healthy controls.

Characteristic	IBS cases (*N* = 163)	Healthy controls (*N* = 194)	Parasite carriage OR (95% CI)	Total subjects (*N* = 357)
Any parasite (*N*)	27	30	1.09 (0.62–1.91)	57
Pathogenic parasite (*N*)	9	8	1.36 (0.51–3.61)	17
Individual parasites (*N*)				
*B. hominis * ^†^ *G. lamblia * ^‡^ *E. coli * *E. histolytica * ^‡^ */dispar * *I. butschlii * *E. nana * *C. mesnili * *T. trichiura * ^‡^ *A. lumbricoides * ^‡^ *H. nana * ^‡^	13 5 15 18 7 9 5 3 1 1	20 7 16 26 6 7 1 1 0 0	0.75 (0.36–1.57) 0.85 (0.26–2.72) 1.14 (0.55–2.39) 0.81 (0.43–1.53) 1.41 (0.46–4.27) 1.56 (0.57–4.29) 6.12 (0.71–52.82) 3.62 (0.37–35.13) — —	33 12 31 44 13 16 6 4 1 1

(1) Mantel-Haenszel odds ratios (ORs) were calculated to determine the association between IBS and parasite infection. Factors such as age, gender, poverty index, household water source, latrine or toilet use, and neighborhood were not confounders and were not included in the final model.

(2) Individuals may have been infected with more than one parasite.

(3) Individual parasites include *Blastocystis hominis, Giardia lamblia, Entamoeba coli, Entamoeba histolytica, Entamoeba dispar, Iodamoeba butschlii, Endolimax nana, Chilomastix mesnili, Trichuris trichiura*, *Ascaris lumbricoides, Hymenolepis nana.*

^†^Pathogenicity depends on parasite load; classified as commensal for the analysis.

^‡^Denotes pathogen.
